# Quercetin Pretreatment Attenuates Hepatic Ischemia Reperfusion-Induced Apoptosis and Autophagy by Inhibiting ERK/NF-*κ*B Pathway

**DOI:** 10.1155/2017/9724217

**Published:** 2017-10-16

**Authors:** Liwei Wu, Qinghui Zhang, Weiqi Dai, Sainan Li, Jiao Feng, Jingjing Li, Tong Liu, Shizan Xu, Wenwen Wang, Xiya Lu, Qiang Yu, Kan Chen, Yujing Xia, Jie Lu, Yingqun Zhou, Xiaoming Fan, Chuanyong Guo

**Affiliations:** ^1^Department of Gastroenterology, Shanghai Tenth People's Hospital, Tongji University School of Medicine, Shanghai 200072, China; ^2^Department of Clinical Laboratory, Kunshan First People's Hospital Affiliated to Jiangsu University, Kunshan, Jiangsu 215300, China; ^3^Department of Gastroenterology, Shanghai Tenth People's Hospital, School of Clinical Medicine of Nanjing Medical University, Shanghai 200072, China; ^4^Department of Gastroenterology, Jinshan Hospital of Fudan University, Jinshan, Shanghai 201508, China

## Abstract

**Background:**

Hepatic ischemia reperfusion (IR) injury is a common phenomenon in transplantation or trauma. The aim of the present study was to determine the protective effect of quercetin (QE) on hepatic IR injury via the ERK/NF-*κ*B pathway.

**Methods:**

Mice were randomized into the sham, IR, QE100 + IR, and QE200 + IR groups. Quercetin was administered intragastrically daily at two doses (100 mg/kg and 200 mg/kg) for 5 days prior to IR injury. The expression levels of liver enzymes, inflammatory cytokines, and other marker proteins were determined at 2, 8, and 24 hours after IR. And they were compared among these groups.

**Results:**

Compared with the IR group, the treatment of QE reduced the release of cytokines, leading to inhibition of apoptosis and autophagy via downregulation of the ERK/NF-*κ*B pathway in this model of hepatic IR injury.

**Conclusion:**

Apoptosis and autophagy caused by hepatic IR injury were inhibited by QE following a reduction in the release of inflammatory cytokines, and the relationship between the two may be associated with inactivation of the ERK/NF-*κ*B pathway.

## 1. Introduction

Ischemia reperfusion (IR) injury, the interruption of blood supply to an organ (ischemia) and its subsequent restoration (reperfusion), leads to irreversible damage. Transient episodes of liver IR always occur during liver transplantation, surgical liver resection, trauma, and hypovolemic shock [1, 2]. One of key factors in limiting progress in hepatobiliary surgery is whether doctors can avoid hepatic IR injury. During transplantation, IR damage is maintained during cold storage of the graft in preservation solution after explantation from the donor and during following reperfusion and implantation into the recipient [3]. In the field of transplantation, IR injury has a tight relationship with the development of acute rejection, primary graft nonfunction, delayed graft dysfunction or poor function, late graft failure, and subsequent graft rejection. Totally speaking, IR injury is tightly associated with the quality and function of the graft following liver transplantation and is the main cause of increased length of hospital stay and decreased long-term graft survival [4, 5]. Therefore, a protective strategy against liver IR injury is urgently needed.

Hepatic IR injury is commonplace in liver surgery, and the underlying mechanism responsible for this injury is diverse and complex. A large number of factors and mediators play important roles in IR injury [6]. During the IR process, the occlusion of blood vessels causes oxygen deficiency in the liver and oxygenated blood reperfusion aggravates the ischemia injury, which is mainly characterized by the depletion of adenosine triphosphate (ATP). The shortage of ATP results in a perturbation of calcium homeostasis and cell swelling. Kupffer cells play a key role in hepatic IR injury and tumor necrosis factor- (TNF-) *α* is a central mediator. In the initial phase of reperfusion, Kupffer cells are activated and induce oxidative stress. Kupffer cells release a large number of proinflammatory cytokines, such as tumor TNF-*α*, interleukin- (IL-) 1, and IL-6, as well as reactive oxygen species (ROS). These actions lead to the accumulation of neutrophils in the liver and the release of inflammatory mediators, ROS, and several proteases, causing hepatocyte damage [7, 8]. TNF-*α* and many other mediators can activate other proteins involved in apoptosis, and many studies have shown that suppression of apoptosis is beneficial for survival after IR [8–12].

Autophagy is involved in both healthy and diseased liver [13–15] and is an intracellular self-digesting pathway, which can be divided into three main types: chaperone-mediated autophagy, microautophagy, and macroautophagy [16, 17]. The expression of Beclin-1, microtubule-associated protein 1 light chain 3 (LC3), and P62 are regarded as markers of autophagy. Although studied for many years, the role of autophagy in hepatic IR injury remains controversial. Some researchers have suggested that autophagy protects hepatocytes and reduces liver IR injury [18–20]; however, others suggest that an increased level of autophagy aggravates damage in liver tissues after IR [21–23]. Therefore, it is necessary to determine whether autophagy protects against or promotes liver injury following IR.

As proinflammatory cytokines, apoptosis, and autophagy play critical roles in hepatic IR injury, these factors should be carefully considered when choosing a drug to treat IR. Quercetin (3,3′,4′,5,7-pentahydroflavone, QE), a member of the flavonoid family, can be found in many types of fruits and vegetables [24] and has many beneficial characteristics, including anti-inflammation, antitumor, and antioxidation activity [25–27]. These properties have been confirmed by many animal models, including ConA-induced hepatitis [28], cholestatic liver injury [29], radiation-induced lung injury [30], carbon tetrachloride- (CCl_4_-) induced liver injury [31], acute pancreatitis [32], and acute myocardial infarction [33]. It was also reported by Arikan et al. that QE protected rats from retina IR injury by reducing apoptosis [34]. In addition, Ghosh et al. found that the antioxidant and antiapoptosis effect of QE was critical in combating IR-induced neuronal damage [35]. However, the effects of QE in hepatic IR injury remain unclear. In addition, it is unknown whether apoptosis and autophagy are involved and their possible mechanisms have not been fully clarified.

Extracellular signal-regulated kinase (ERK), which was discovered over 30 years ago, belongs to the family of mitogen-activated protein kinases (MAPKs) [36]. MAPKs are composed of three conserved and sequentially acting kinases: MAPK, MAPK kinase (MAPKK), and MAPKK kinase (MAPKKK). The ERK pathway acts via the activation of Raf (MAPKKK), which phosphorylates and activates MEK (MAPKK), which then activates ERK (MAPK) through phosphorylation and dissociates from ERK [37, 38]. The ERK pathway can be activated by a wide variety of stimuli, including cytokines, cell stress, hormones, drugs, toxins, and metabolic changes. Nuclear factor- (NF-) *κ*B, is a member of the Rel family, which is composed of c-Rel, RelA (p65), RelB, p50, and p52 [39]. NF-*κ*B regulates a number of genes, especially those involved in immune and inflammation response. As the function and action of ERK and NF-*κ*B have many common points and some studies have shown that ERK and NF-*κ*B have a close relationship in the inflammatory response [40–42], we speculated that the ERK/NF-*κ*B pathway may also protect liver tissue from injury.

In the present study, we aimed to elucidate the protective mechanisms of QE on hepatic IR injury. It is hypothesized that pretreatment with QE can attenuate apoptosis and autophagy induced by IR via the ERK/NF-*κ*B pathway.

## 2. Materials and Methods

### 2.1. Reagents

Quercetin (QE), dimethyl sulfoxide (DMSO) and collagenase, and D-Hanks buffer were purchased from Sigma-Aldrich (St. Louis, MO, USA). Aspartate aminotransferase (AST) and alanine aminotransferase (ALT) microplate test kits used in this study were purchased from Jiancheng Bioengineering Institute (Jiancheng Biotech, Nanjing, China). Enzyme-linked immunosorbent assay (ELISA) kits for TNF-*α* and IL-6 were purchased from Anogen (Canada). 3-(4,5)-Dimethylthiahiazo (-zy1)-3,5-di-phenytetrazoliumromide (MTT) was purchased from Peptide Institute Inc. (Peptide Institute Inc., Osaka, Japan). The antibodies used in this study included TNF-*α*, IL-6, Bcl-2, LC-3, caspase 3, and NF-*κ*B (all from Cell Signaling Technology, Danvers, MA, USA); caspase 9, Bax, Beclin-1, and P62 (all from Proteintech, Chicago, IL, USA); and ERK and p-ERK (both from Epitomics, Burlingame, CA, USA). The PCR kit was from Takara (Takara Biotechnology, Dalian, China).

### 2.2. Hepatocyte Isolation and Drug Preparation

Primary hepatocytes were isolated by a modified in situ collagenase perfusion technique as what previous studies described [43]. Briefly, the portal vein was cannulated after anesthesia and laparotomy. The liver was perfused with 10 mL of prewarmed D-Hanks buffer for 10 min and then 5 mL of 0.02% type V collagenase solution. After perfusion, liver tissues were removed and washed with 20 mL D-Hanks buffer. Hepatic tissues were dispersed and incubated in 20 mL of 0.01% collagenase in a 37°C shaking water bath for about 30 min. The cell suspensions were then filtered into a glass tube and centrifuged at 800*g* for 5 min and washed with Roswell Park Memorial Institute- (RPMI-) 1640 culture medium (Thermo, China) and finally incubated at 37°C with 5% CO2.

Quercetin was solubilized in DMSO at a concentration of 10 mM, stored at 4°C, and protected from light. Different concentrations of quercetin were prepared before used and added to cells. In all experiments, the concentration of DMSO never exceeded 1%.

### 2.3. Cell Culture and Cell Proliferation and Viability

Primary hepatocytes were cultured in RPMI-1640 culture medium supplemented with 10% fetal bovine serum (Hycione, South America), 100 mg/mL streptomycin (Gibco, Canada), and 100 U/mL penicillin in a humidified incubator at 37°C in 5% CO2. Hepatocyte purity and viability typically exceeded 99% and 95%, respectively.

Primary hepatocytes were plated at a 2 × 10^4^ cell/well in 96-well plates(100 *μ*L of medium per well). After seeding for one day, hepatocytes were treated with QE (5, 10, 20, 30, and 40 *μ*M) for 24 hours. The cells were then treated with AR, which means hypoxia (3% O_2_, 5% CO_2_, and 92% N_2_) for 24 h and reoxygenation (5% CO_2_, 95% air) for 2 h. Cell viability was measured with the CCK8 assay at a wavelength of 450 nm. The experiments were repeated three times.

Primary hepatocytes were divided into groups: (1) control group, no treatment; (2) QE group, treated with quercetin diluted in DMSO at a concentration of 20 *μ*M; (3) DMSO group, treated with DMSO; (4) AR group, treated with AR injury without any treatment; (5) AR + QE5 group, 5 *μ*M quercetin administrated 24 h before AR; (6) AR + QE10 group, 5 *μ*M quercetin administrated 24 h before AR; and (7) AR + QE20 group, 20 *μ*M quercetin administrated 24 h before AR.

### 2.4. Apoptosis Detected by Flow Cytometry

Primary hepatocytes were plated in six-well plates. Cells in the control group, AR group, and AR + QE20 group were collected, washed twice in ice-cold PBS, and mixed with 100 *μ*L of 1× binding buffer. Annexin V-fluorescein isothiocyanate (FITC) and propidium iodide (PI) solution were added, and cells were incubated at room temperature for 15 min. Cell apoptosis was assessed with FITC (BD Pharmingen, San Jose, CA, USA). A flow cytometric analysis was performed on cells that were in the early apoptosis (annexin V+/PI−) or late apoptosis/necrosis (annexin V+/PI+) phase.

### 2.5. Animals and Surgical Procedures

Male Balb/c mice (7-8 weeks old, 23 ± 2 g) were purchased from Shanghai SLAC Laboratory Animal Co. Ltd. (Shanghai, China). The mice were raised in a clean environment, which was maintained at a temperature of 24°C ± 2°C and 55% humidity under a 12 : 12 hour light : dark cycle, and the animals had free access to food and water. All animal experiments were performed in accordance with the National Institutes of Health Guidelines and were approved by the Animal Care and Use Committee of Shanghai Tongji University.

A mouse model of 70% hepatic warm ischemia was established based on a previously reported method [44–46]. Mice were fasted for approximately 24 hours but allowed water before surgery. The mice were anesthetized with 1.25% sodium pentobarbital (Nembutal, St. Louis, MO, USA) by intraperitoneal injection. After that, the mice were placed on a sterile and heated experimental table and a midline laparotomy was performed on each animal. Blood flow to the left lateral and median liver lobes was occluded for 45 minutes using a metal microvascular clamp to achieve hepatic ischemia. Continuous reperfusion was then achieved by loosening the clamps. The abdominal cavity was then closed. To maintain a constant body temperature, we used animal body temperature maintenance instructions (ZS Dichuang, Beijing, China).

### 2.6. Drug Treatment

QE was diluted with 0.9% saline to 12.5 mg/ml (for QE100) and 25 mg/ml (for QE200) and was administered intragastrically 0.2 ml once per day for 5 consecutive days [47]. Mice were randomly divided into the following eight groups:

1. Natural control group (*n* = 5): mice received no treatment
2. Vehicle group (*n* = 5): mice received 0.9% saline by gavage once a day for 5 days
3. Low-QE group (*n* = 5): mice received 100 mg/kg QE by gavage once a day for 5 days
4. High-QE group (*n* = 5): mice received 200 mg/kg QE by gavage once a day for 5 days
5. Sham group (*n* = 24): mice received 0.9% saline alone, without IR, and underwent sham operation
6. IR group (*n* = 24): mice received 0.9% saline by gavage before IR
7. Low-QE + IR group (*n* = 24): mice received 100 mg/kg QE by gavage before IR
8. High-QE + IR group (*n* = 24): mice received 200 mg/kg QE by gavage before IR.

For the first four groups, mice were sacrificed after five days, and for the other four groups, eight mice were randomly selected from each group and sacrificed at 2, 8, and 24 hours after IR. Serum and liver tissues were collected for analysis, including ALT, AST, cytokine levels, and pathological changes.

### 2.7. Biochemical Analysis

After taken from their hearts, blood samples were placed at 4°C for 4-5 hours, and then, serum was separated by centrifuging at 4600 ×g for 10 minutes, finally storing at −80°C. The levels of serum ALT and AST were measured using an automated chemical analyzer (Olympus AU1000, Olympus, Tokyo, Japan). The plasma levels of TNF-*α* and IL-6 were measured using ELISA kits following the appropriate protocols.

### 2.8. Histopathology

A section of the left liver lobe was incubated in 4% paraformaldehyde for more than 24 hours and then dehydrated using different concentrations of ethyl alcohol. The specimen was then embedded in paraffin. Sections were cut at a thickness of 5 *μ*m and stained with hematoxylin and eosin (H&E), which were observed under light microscopy to determine tissue damage and inflammatory level.

### 2.9. Western Blotting

Hepatic tissues and primary hepatocytes were homogenized in radioimmunoprecipitation assay (RIPA) lysis buffer with phenylmethane-sulfonyl fluoride (PMSF) and protease inhibitors (PI). The concentration of tissue protein was calculated using the bicinchoninic acid protein assay (BCA) (Kaiji Biology, Nanjing, China). Protein samples then were mixed with 5× sodium dodecyl sulfate-polyacrylamide gel electrophoresis (SDS-PAGE) sample loading buffer and boiled at 100°C for 10 minutes. The protein samples were then stored at −20°C. Protein samples were separated on 10% or 12.5% sodium dodecyl sulfate polyacrylamide gels and transferred onto a nitrocellulose (NC) filter membrane or a polyvinylidene difluoride (PVDF) membrane. Nonspecific binding sites on membranes were blotted with 5% nonfat milk for more than 1 hour and incubated with primary antibodies at 4°C overnight. The primary antibodies used were *β*-actin (1 : 1000), TNF-*α* (1 : 500), IL-6 (1 : 500), Bax (1 : 1000), Bcl-2 (1 : 1000), Beclin-1 (1 : 1000), LC3 (1 : 500), P62 (1 : 1000), caspase 3 (1 : 500), caspase 9 (1 : 500), ERK (1 : 1000), p-ERK (1 : 500), and NF-*κ*B (1 : 500). The following day, the membranes were washed in PBST (1 mL Tween in 1 L PBS) 3 times (10 minutes each time). The membranes were incubated with secondary goat anti-mouse or anti-rabbit antibodies at room temperature for 1 hour. After washing in PBST for 30 minutes, the membranes were scanned using the Odyssey two-color infrared laser imaging system.

### 2.10. Immunohistochemistry

The prepared sections (5 *μ*m) were heated at 60°C for 1 hour and then dewaxed and dehydrated with xylene and different concentrations of alcohol. Antigens were drenched in citrate buffer (pH 6.0) and treated with an antigen retrieval technique by heating in a water bath at 95°C for 10 minutes and cooling to room temperature. To block the activity of endogenous peroxidase, the sections were covered in hydrogen peroxide solution (3%) for 20 minutes at 37°C and then washed three times in phosphate buffer solution (PBS). In order to block the nonspecific binding sites, the sections were immersed in 5% bovine serum albumin (BSA) at 37°C for 20 minutes and then incubated at room temperature for 10 minutes. The slices were then incubated overnight at 4°C with TNF-*α* (1 : 100), IL-6 (1 : 100), Bax (1 : 100), Bcl-2 (1 : 100), P62 (1 : 100), Beclin-1 (1 : 500), LC3 (1 : 500), NF-*κ*B (1 : 200), and p-ERK (1 : 100). On the second day, the sections were incubated with a secondary antibody for 30 minutes and were analyzed with a diaminobenzidine kit. The slices were then observed under a light microscope. The integrated optical densities (IODs) of different indices were calculated using Image-Pro Plus software 6.0 (Media Cybernetics, Silver Spring, MD, USA).

### 2.11. Terminal Deoxynucleotidyl Transferase dUTP Nick End Labeling (TUNEL) Assay

Apoptosis in liver tissue was measured by the TUNEL assay (Roche, Mannheim, Germany) following the appropriate protocol. The sections were deparaffinized and digested with 20 *μ*g/mL proteinase K at room temperature for 15 minutes. The sections were then incubated in the TUNEL reaction mixture at 37°C for 1 hour. TUNEL-positive cells were observed and counted under light microscopy.

### 2.12. Reverse Transcription- (RT-) PCR and Quantitative Real-Time- (qRT-) PCR

Total RNA in liver tissue was isolated with TRIzol and then reverse transcribed into cDNA using the reverse transcription kit protocol (Takara Biotechnology, Dalian, China). Target gene expression was detected by SYBR Green Quantitative RT-PCR using a 7900HT fast real-time PCR system (Applied Biosystems, Foster City, CA, USA).  Table 1 shows the primer sequences used in this study.

### 2.13. Statistical Analysis

All data are shown as mean ± standard deviation (SD). ELISA and qRT-PCR data were analyzed by one-way analysis of variance. The necrotic or edematous areas, Western blotting results, and serum ALT and AST levels were analyzed using the Student's *t*-test. *P* < 0.05 was considered statistically significant. All figures were designed using Graphpad Prism software (v 6.0).

## 3. Results

### 3.1. QE and Surgery Had No Effect on Normal Liver Tissue

**Figure 1:**
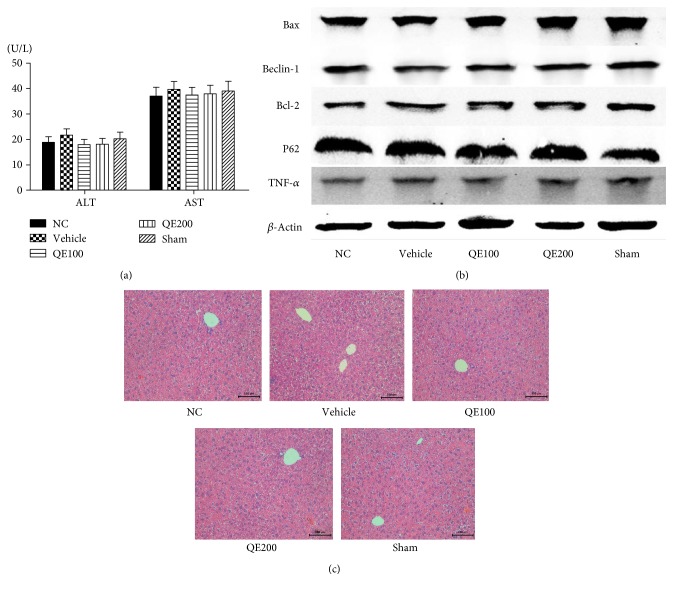
The effects of QE on normal mice. Notes: (a) the levels of serum ALT and AST in the five groups did not differ. Data are given as means ± SD (*n* = 5, *P* > 0.05). (b) Western blots of the expression of Bax, Bcl-2, Beclin-1, P62, and TNF-*α*. (c) Representative H&E-stained sections of the liver (original magnification, ×200).

To ensure that the differences between the IR group and the pretreatment groups were all due to QE, we first measured the influence of QE and surgery on normal liver tissue. Liver function, pathology, and markers related to damage (Bax, Beclin-1, Bcl-2, and P62) and inflammation (TNF-*α*) were determined. The results showed that the serum level of liver enzymes and the expression of marker proteins in the 5 groups were not obviously different (Figures 1(a) and 1(b)).  Figure 1(c) also shows that there were no pathological and morphological changes in the 5 groups. These findings confirmed that QE and surgery did not affect normal liver tissue.

### 3.2. QE Pretreatment Ameliorated Hepatic IR Injury

**Figure 2:**
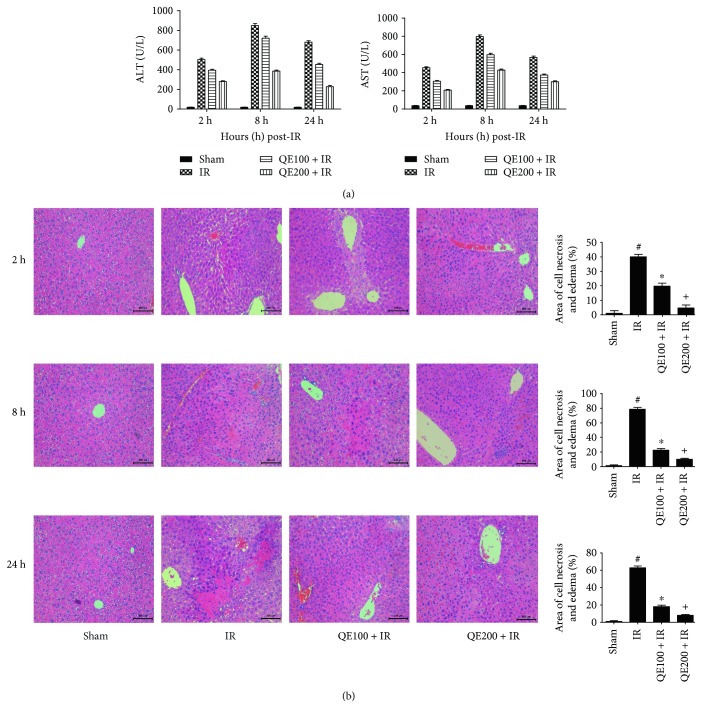
Effects of QE on hepatic ischemia-reperfusion injury. Notes: (a) the levels of serum ALT and AST changed depending on the quercetin dose, 100 mg/kg or 200 mg/kg. Data are given as means ± SD (*n* = 8, ^∗^*P* < 0.05 for sham versus IR, ^#^*P* < 0.05 for QE100 + IR versus IR, and ^+^*P* < 0.05 for QE200 + IR versus IR). (b) Representative H&E-stained sections of the liver were analyzed with Image-Pro Plus 6.0 (original magnification, ×200). The results show statistically significant differences among the different groups (*n* = 8, ^∗^*P* < 0.05 for sham versus IR, ^#^*P* < 0.05 for QE100 + IR versus IR, and ^+^*P* < 0.05 for QE200 + IR versus IR).

ALT and AST are sensitive indicators of liver damage and may increase rapidly after damage. To assess the effects of QE on hepatic IR injury, we measured the levels of ALT and AST in sera. The results in Figure 2(a) show that these enzymes increased significantly in the IR group compared with the sham group at 2, 8, and 24 hours after reperfusion and got a peak at 8 hours. There were obvious reductions in ALT and AST levels in the QE groups (100 mg/kg and 200 mg/kg) that were dose-dependent. These findings indicated that the liver got the most serious damage at 8 hours after reperfusion, and QE protected liver function in a dose-dependent manner. To confirm these findings, we performed H&E staining to observe pathological changes in the liver. Necrosis areas were characterized by changes of nucleus (pyknosis, karyorrhexis, and karyorrhexis) and complete destruction of cell structure, which showed as the enhancements of cytoplasm eosinophilic. The structure of liver tissues was unaffected in the sham group; however, disorganized liver structure, with complete destruction of cell structure; disordered lobular structure; and marked necrosis were observed in the IR group at 2, 8, and 24 h. The necrotic areas were reduced in the QE groups, and the high-dose QE group showed a greater effect (Figure 2(b)).

### 3.3. QE Reduced the Release of Proinflammatory Factors Including TNF-*α* and IL-6

**Figure 3:**
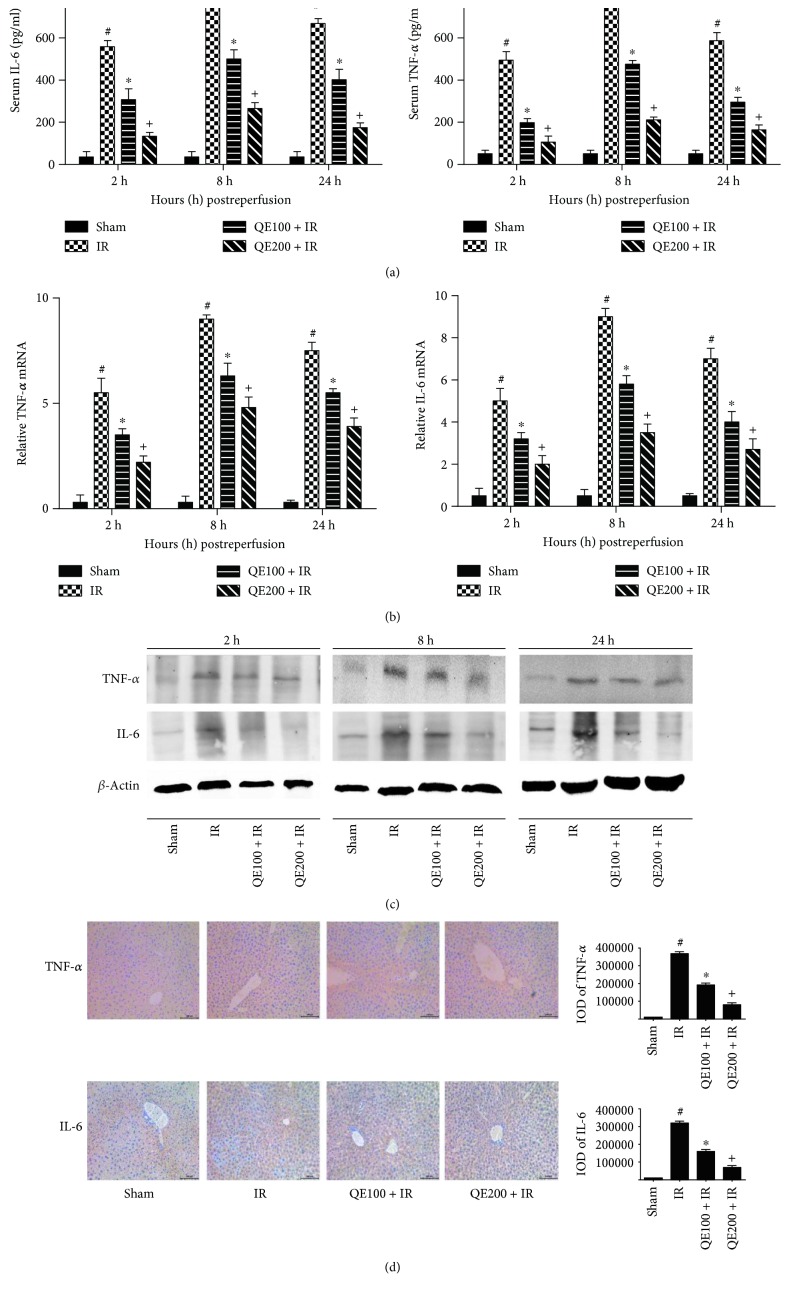
Effects of QE on the release of TNF-*α* and IL-6 in mice on hepatic ischemia-reperfusion injury. Notes: (a) the levels of serum TNF-*α* and IL-6 measured with ELISAs were reduced by quercetin pretreatment in mice at doses of 100 mg/kg and 200 mg/kg. Data are presented as means ± SD (*n* = 8, ^∗^*P* < 0.05 for sham versus IR, ^#^*P* < 0.05 for QE100 + IR versus IR, and ^+^*P* < 0.05 for QE200 + IR versus IR). (b) The mRNA levels of TNF-*α* and IL-6 were evaluated in each group with qRT-PCR (*n* = 8, ^∗^*P* < 0.05 for sham versus IR, ^#^*P* < 0.05 for QE100 + IR versus IR, and ^+^*P* < 0.05 for QE200 + IR versus IR). (c) The protein expression levels of the TNF-*α* and IL-6 were determined with Western blotting by grey bands at each time point. (d) Immunohistochemistry staining (×200) showing the expression of TNF-*α* and IL-6 in liver tissue at 8 h. The ratio of brown area to total area was analyzed with Image-Pro Plus 6.0 (*n* = 8, ^∗^*P* < 0.05 for sham versus IR, ^#^*P* < 0.05 for QE100 + IR versus IR, and ^+^*P* < 0.05 for QE200 + IR versus IR).

Inflammation is an important cause of liver IR injury. Proinflammatory cytokines were detected to further understand the mechanism of IR injury. Therefore, we performed ELISA, real-time PCR, Western blotting, and histochemical staining to determine TNF-*α* and IL-6 levels and the results are shown in Figure 3. The expression of TNF-*α* and IL-6 increased in both plasma and liver tissues. The ELISA results showed that the levels of TNF-*α* and IL-6 increased in the IR group and peaked at 8 hours after reperfusion. In addition, QE reduced the release of TNF-*α* and IL-6 (Figure 3(a)). Compared with the sham group, the gene and protein levels of TNF-*α* and IL-6 clearly increased in the IR group. Quercetin pretreatment markedly attenuated the increased levels of TNF-*α* and IL-6 (Figures 3(b) and 3(c)). We chose liver tissues at 8 hours after reperfusion for immunohistochemical staining, and the results were in accordance with the results of mRNA and protein expression mentioned above (Figure 3(d)). (The immunohistochemical staining results of 2 and 24 hours were shown in Figures S1 and S2 available online at https://doi.org/10.1155/2017/9724217). Besides, we found more inflammation cell infiltration in H&E staining for IR group than that for QE groups. In summary, these results showed that QE inhibited the production of proinflammatory cytokines, such as TNF-*α* and IL-6 in serum and liver tissue.

### 3.4. QE Pretreatment Alleviated Apoptosis in Hepatic IR Injury

**Figure 4:**
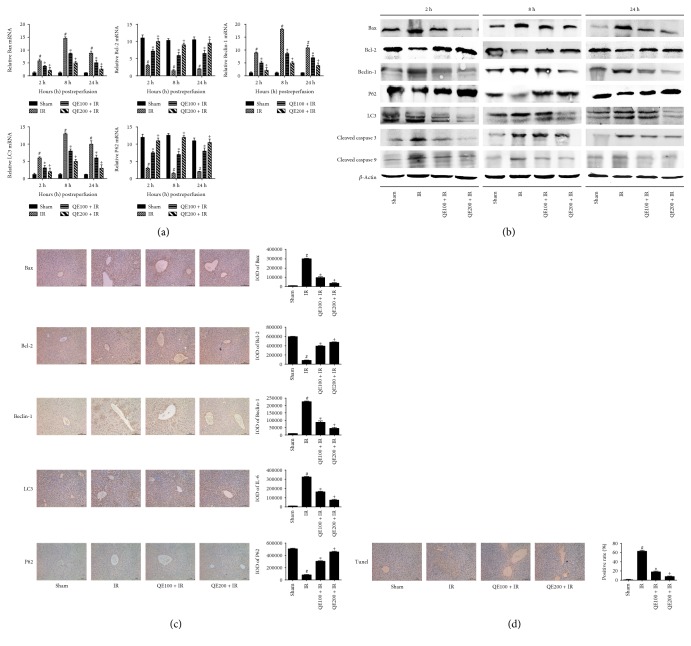
Effects of QE on apoptosis and autophagy in mice on hepatic ischemia-reperfusion injury. Notes: (a) mRNA levels of Bax, Bcl-2, Beclin-1, LC3, and P62 were measured with real-time PCR (*n* = 8, ^∗^*P* < 0.05 for sham versus IR, ^#^*P* < 0.05 for QE100 + IR versus IR, and ^+^*P* < 0.05 for QE200 + IR versus IR). (b) Protein expression of Bax, Bcl-2, Beclin-1, LC3, P62, caspase 3, and caspase 9 were detected with Western blotting. (c) Immunohistochemistry staining (×200) showed the expression of Bax, Bcl-2, Beclin-1, P62, and LC3 protein in liver tissues at 8 h. The ratio of positive area to total area was counted with Image-Pro Plus (v 6.0) (*n* = 8, ^∗^*P* < 0.05 for sham versus IR, ^#^*P* < 0.05 for QE100 + IR versus IR, and ^+^*P* < 0.05 for QE200 + IR versus IR). (d) TUNEL staining (×200) showed apoptotic cells in three groups at 8 hours. The percentage of TUNEL-positive cells are expressed as means ± SD (*n* = 8, ^∗^*P* < 0.05 for sham versus IR, ^#^*P* < 0.05 for QE100 + IR versus IR, and ^+^*P* < 0.05 for QE200 + IR versus IR).

Previous studies have reported the participation of apoptosis in hepatic IR. Bcl-2, Bax, caspase 3, and caspase 9 are known to regulate apoptosis. Thus, to study the possible protective mechanism of QE on apoptosis during hepatic IR, we determined the levels of these factors. As shown in Figure 4(a), the mRNA levels of Bcl-2, an antiapoptotic protein, clearly increased in the QE + IR groups compared with the IR group, and the mRNA levels of proapoptotic proteins, Bax, caspase 3, and caspase 9, were obviously increased in the IR group compared with the QE + IR groups. To confirm the positive effects of QE, we examined the protein levels of these factors (Figure 4(b)). We observed increased expression of Bax, caspase 3, and caspase 9 and decreased expression of Bcl-2 in the IR group compared with the QE pretreatment groups. In addition, similar results were observed for immunohistochemistry staining (Figure 4(c)). (The immunohistochemical staining results of 2 and 24 were shown in Figures S1 and S2). Apoptotic cells were also detected by TUNEL staining. As shown in Figure 4(d), numerous TUNEL-positive cells, whose nucleus was dyed dark grown, were observed in the IR group and the number in the QE + IR group was markedly decreased. These results demonstrated that QE had a good effect on IR-induced apoptosis.

### 3.5. QE Pretreatment Inhibited Autophagy in Hepatic IR Injury

**Figure 5:**
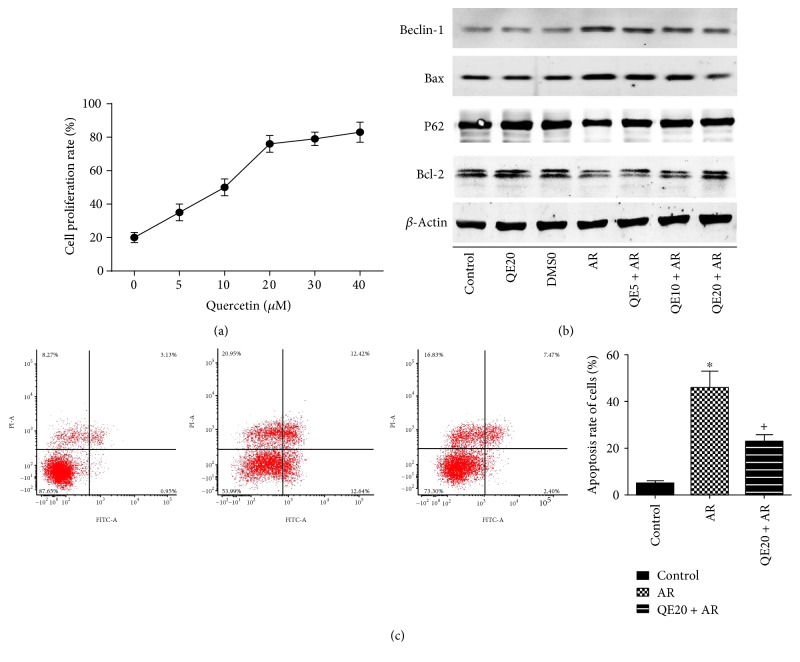
Effects of QE on the proliferation and apoptosis of primary hepatocytes induced by AR. Notes: (a) the proliferation of primary hepatocytes treated with QE before AR induction was detected using CCK8. (b) The protein levels of Beclin-1, P62, Bcl-2, and Bax in primary hepatocytes were detected by Western blot. (c) The apoptosis of primary hepatocytes was determined by flow cytometry (*n* = 3, ^∗^*P* < 0.05 for control versus AR, ^+^*P* < 0.05 for QE20 + AR versus AR).

To evaluate the effects of QE on autophagy, we measured the levels of LC3, Beclin-1, and P62 by Western blotting, real-time PCR, and immunohistochemistry. The mRNA and protein levels of LC3, Beclin-1, and P62 are shown in Figures 4(a) and 4(b). When compared with the IR group, the expression of P62 was increased and the expression of Beclin-1 and LC3 was decreased in the QE pretreatment groups. As shown in Figure 4(c), the ratio of positive areas of LC3, Beclin-1, and P62 shown by immunohistochemistry were concordant with the results of gene and protein expression levels. (The immunohistochemical staining results of 2 and 24 hours were shown in Figures S1 and S2) Besides, we got consistent results in vitro experiment, which were shown in Figure 5(b). These results suggested that QE had a beneficial effect on IR-induced autophagy.

### 3.6. QE Attenuates IR Injury by Inhibiting the ERK/NF-*κ*B Pathway

**Figure 6:**
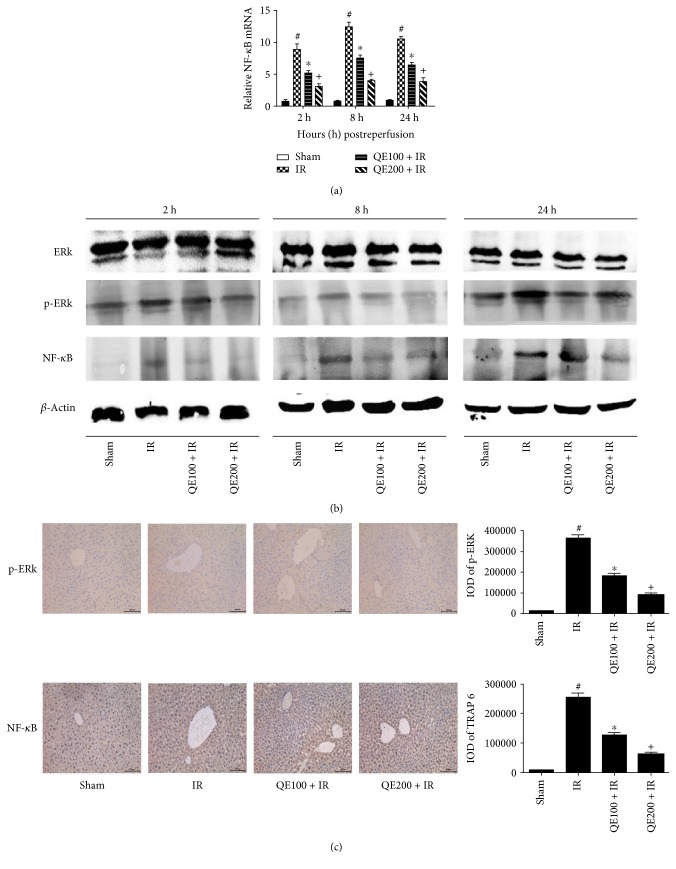
Effects of QE on the regulation of the ERK/NF-*κ*B pathway in mice on hepatic ischemia-reperfusion injury. Notes: (a) the mRNA level of NF-*κ*B was detected by qRT-PCR (*n* = 8, ^∗^*P* < 0.05 for sham versus IR, ^#^*P* < 0.05 for QE100 + IR versus IR, and ^+^*P* < 0.05 for QE200 + IR versus IR). (b) The expression of ERK, p-ERK, and NF-*κ*B was determined by Western blotting. (c) Immunohistochemistry staining (×200) showed the expression of p-ERK and NF-*κ*B protein in liver tissues at 8 h. The ratio of positive area to total area was counted with Image-Pro Plus (v 6.0) (*n* = 8, ^∗^*P* < 0.05 for sham versus IR, ^#^*P* < 0.05 for QE100 + IR versus IR, and ^+^*P* < 0.05 for QE200 + IR versus IR).

Inflammation pathways play an essential role in hepatic IR injury. Therefore, the expression of ERK and NF-*κ*B was detected for further determination of the mechanism of QE. As shown in Figure 6(a), no difference in total ERK expression was observed between the sham, IR, and QE groups. Expression of the activated ERK form, phosphorylated ERK (p-ERK), was significantly increased in the IR group, compared with the sham group; however, the p-ERK level was decreased in the QE pretreatment group. The results in Figures 6(a) and 6(b) show that the gene and protein expression of NF-*κ*B was overtly upregulated in the IR group compared with the sham group and was downregulated in the QE groups. Similar results were obtained for immunohistochemistry staining (Figure 6(c)). (The immunohistochemical staining results of 2 and 24 hours were shown in Figures S1 and S2). In conclusion, we speculate that QE attenuated IR injury, at least partly, by inhibition of the ERK/NF-*κ*B pathway.

### 3.7. Quercetin Protected the Proliferation of Primary Hepatocyte Induced by AR and Inhibited Their Apoptosis

Hepatocyte proliferation was measured by using CCK8. The results of Figure 5(a) showed that after treated with increasing concentrations (0–40 *μ*M) of quercetin before AR damage, primary hepatocytes proliferated dose-dependently. Then, we chose 5, 10, and 20 *μ*M as effective dose of quercetin for Western blotting and 20 *μ*M for flow cytometry. Results for Western blotting (Figure 5(b)) and flow cytometry (Figure 5(c)) showed that hepatocytes appeared obviously at apoptosis after AR damage, while quercetin pretreatment attenuated that effect, which indicated that quercetin protected primary hepatocytes from AR-induced cell injury.

## 4. Discussion

Liver IR injury occurs in many clinical situations in surgery and is linked with increased morbidity and mortality. However, due to the complex underlying mechanism of hepatic IR, an effective strategy to protect against IR is unavailable. Quercetin, which is a member of the flavonoid family, has obvious anti-inflammation and antiapoptosis properties. As it has been demonstrated that inflammation, apoptosis, and autophagy are main factors in IR damage, we hypothesized that QE could protect the liver from IR damage.

In our study, hepatic ischemia for 45 minutes followed by reperfusion for 2, 8, and 24 hours led to serious liver dysfunction. The extent of liver damage after IR is usually assessed by increased serum levels of liver enzymes, mainly ALT and AST. In addition, the release of proinflammatory cytokines and the gene and protein expression of apoptosis and autophagy markers significantly increased in the IR group. Compared with the IR group, ALT and AST levels in the QE groups decreased in a dose-dependent manner, which indicated that QE protected the liver against injury. As the inflammatory response is a very important part of IR injury, we first determined the protein and gene levels of the proinflammatory cytokines, TNF-*α* and IL-6. The results were consistent with our hypothesis that IR injury stimulated the sharp release of TNF-*α* and IL-6 and pretreatment with QE significantly decreased the release of TNF-*α* and IL-6. TNF-*α* has been proved to be an important mediator of apoptosis and necrosis via TNF-receptor 1 [9] and can activate many signaling pathways, for example, MAPKs, NF-*κ*B, and PI3K/Akt, in addition to IL-6.

Furthermore, to verify the antiapoptosis effect of QE, we carried out qRT-PCR and Western blotting to detect the expression of caspase 3, caspase 9, Bax, and Bcl-2, which are markers of apoptosis. Apoptosis is a typical form of programmed cell death and can be regulated by inflammatory cytokines, such as TNF-*α*. Hepatic apoptosis is a major pathological feature in liver injury [13]. As expected, pretreatment with QE reduced the expression of these proapoptotic proteins and increased the expression of antiapoptosis proteins in a dose-dependent manner. Also, we got similar results in our vitro experiments. Thus, the anti-inflammation and antiapoptosis functions of QE in IR injury were confirmed. We subsequently determined the role of QE in hepatic IR injury.

ERK is a member of the MAPKs and plays a key role in cell signaling and is involved in many aspects of cell physiology, including proliferation, migration, differentiation, and death [48–50]. In quiescent cells, ERK binds with MEK and is localized in the cytoplasm. Once stimulated by extracellular factors, ERK is phosphorylated, dissociates from MEK, and then activates targets such as transcription factors. Some researchers have suggested that ERK is one of the most important mediators in liver injury. The study by Fu et al. showed that acetaminophen-induced liver injury was ameliorated by suppressing the JNK/ERK pathway [51]. Liu et al. found that the ERK pathway can regulate matrix metalloproteinase-1 induction in hepatic stellate cells [52]. The results obtained by Wei et al. demonstrated that by inhibiting the MAPK/ERK pathway, ethanol negatively regulated the differentiation of hECS-derived hepatic progenitors [53]. In addition, Chien et al. found that the metastatic features of HepG2 cells were attenuated by the PKC/ERK pathway [54]. It has also been reported that ERK activity plays a role in energy metabolism [55]. Therefore, we decided to determine the expression of ERK to assess whether it also takes part in IR injury. The results suggested that ERK was closely associated with IR injury. Nuclear factor-kappa B (NF-*κ*B), a transcription factor, plays a critical role both in normal situations and in the harmony of the adaptive immune response. Its subunits can form transcription factors that bind to the promoters of target genes and regulate the expression of many cytokines, such as TNF-*α* and IL-6 [56]. It has been reported that NF-*κ*B may be activated by MAPKs [41, 57].To confirm the mechanism of QE in hepatic IR injury, we determined the expression of ERK, p-ERK, and NF-*κ*B by qRT-PCR, Western blotting, and immunohistochemistry. The expression of ERK was not significantly different between the four groups. However, the IR group showed an obvious increase in the levels of p-ERK and NF-*κ*B, and the levels in the QE groups were significantly decreased compared with the IR group. These results suggested that QE inhibited the activation of ERK, which then blocked the expression of NF-*κ*B. We then measured other proteins to determine how the ERK/NF-*κ*B pathway attenuated liver damage. Firstly, we measured caspase 3, caspase 9, Bax, and Bcl-2. The antiapoptosis effect of Bcl-2 was counteracted by the proapoptotic protein, Bax. The imbalance between Bax and Bcl-2 determines cell survival and death. Therefore, the ratio of Bcl-2 to Bax is a key factor in apoptosis. In our study, QE pretreatment decreased the expression of Bax, caspase 3, and caspase 9 and increased the expression of Bcl-2.

Autophagy is also a critical process in IR injury, and we measured the expression of Beclin-1, LC3, and P62 using Western blotting, qRT-PCR, and immunohistochemistry. Beclin-1 interacts with Bcl-2. When Bcl-2 is inactivated by NF-*κ*B, the Bcl-2-Beclin-1 complex is divided, and free Beclin-1 enhances the induction of autophagy. Microtubule-associated protein 1 light chain 3 (LC3) is first cleaved into LC3 I by autophagy-related gene 4 (Atg4) and then cleaved by Atg5, Atg12, and Atg16 into LC3 II. The transformation of LC3 I to LC3 II contributes to autophagosome formation. Therefore, the LC3 II/LC3 I ratio is usually used as a marker of autophagy. P62 is an autophagy adaptor protein. The results showed that QE pretreatment markedly decreased the expression of Beclin-1 and LC3 and increased P62 expression.

**Figure 7:**
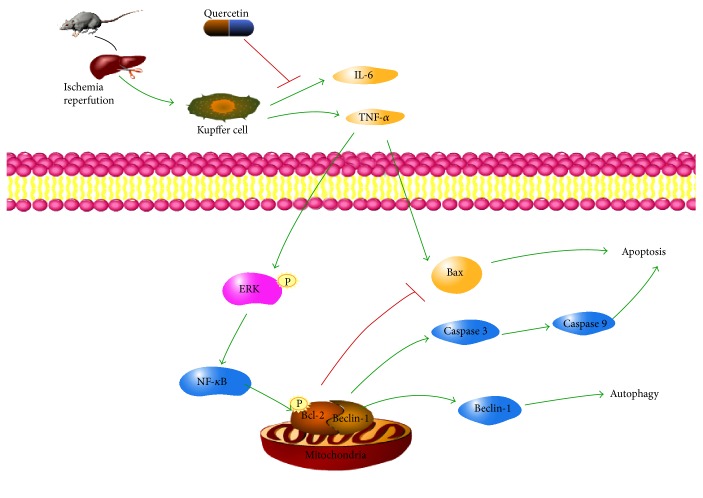
Mechanism of QE action. Notes: in IR-induced hepatitis, QE reduced apoptosis and autophagy by inhibiting ERK/NF-*κ*B pathway. After IR injection, TNF-*α* activated ERK by phosphorylating it, which then led to the excitation of NF-*κ*B. Bcl-2 was phosphorylated, which promoted the release of caspase 9 and caspase 3. They work together with Bax activated by TNF-*α* and led to apoptosis. Beclin-1 dissociated from Bcl-2, enhancing the induction of autophagy. Thus, QE successfully inhibited the release of TNF-*α* in stressed cells during hepatic IR injury and also reduced apoptosis and autophagy by reducing the expression of NF-*κ*B.

Thus, we found that cytokines activated the ERK/NF-*κ*B pathway, which induced apoptosis and autophagy in IR injury, and QE protected the liver from damage (Figure 7). Due to the complex underlying mechanism involved, the relationship between QE and this pathway requires further investigation. Also, the results got from our study showed that quercetin can effectively protect the liver from IR injury and we believed that quercetin could be a promising therapeutic drug. However, whether we can get similar effective treatment results in patients is still uncertain and needs more clinical trials.

## 5. Conclusion

Taken together, our findings showed that quercetin reduced hepatic IR injury in Balb/c mice. Quercetin reduced serum liver enzymes and pathological liver damage. Furthermore, quercetin inhibited the release of inflammatory cytokines, including TNF-*α* and IL-6. The antiapoptotic and antiautophagy effects of quercetin were due to the inhibition of the TNF-*α*-mediated ERK/NF-*κ*B pathway. Our findings suggest that quercetin may be a potential therapeutic agent in the treatment of ischemia reperfusion injury.

## Supplementary Material

Immunohistochemistry for 2 hours after reperfusion. Immunohistochemistry for 24 hours after reperfusion.

## Figures and Tables

**Table 1:** Nucleotide sequences of primers used for qRT-PCR.

Gene		Primer sequence(5′-3′)
TNF-*α*	Forward	CAGGCGGTGCCTATGTCTC
	Reverse	CGATCACCCCGAAGTTCAGTAG
IL-6	Forward	CTGCAAGAGACTTCCATCCAG
	Reverse	AGTGGTATAGACAGGTCTGTTGG
Bax	Forward	AGACAGGGGCCTTTTTGCTAC
	Reverse	AATTCGCCGGAGACACTCG
Bcl-2	Forward	GCTACCGTCGTCGTGACTTCGC
	Reverse	CCCCACCGAACTCAAAGAAGG
Beclin-1	Forward	ATGGAGGGGTCTAAGGCGTC
	Reverse	TGGGCTGTGGTAAGTAATGGA
P62	Forward	GAGGCACCCCGAAACATGG
	Reverse	ACTTATAGCGAGTTCCCACCA
LC3	Forward	GACCGCTGTAAGGAGGTGC
	Reverse	AGAAGCCGAAGGTTTCTTGGG
NF-*κ*B	Forward	ATGGCAGACGATGATCCCTAC
	Reverse	CGGATCGAAATCCCCTCTGTT
*β*-actin	Forward	GGCTGTATTCCCCTCCATCG
	Reverse	CCAGTTGGTAACAATGCCATGT
