# Supplementary material for: Quercetin Pretreatment Attenuates Hepatic Ischemia Reperfusion-Induced Apoptosis and Autophagy by Inhibiting ERK/NF-κB Pathway
Source: Gastroenterol Res Pract. 2017 Oct 16;2017:9724217. doi: 10.1155/2017/9724217 (PMC5662816; doi:10.1155/2017/9724217)

**Quercetin pretreatment attenuates hepatic ischemia reperfusion-induced apoptosis and autophagy by inhibiting ERK/NF-κB pathway**

**Liwei Wu^1^,#,Qinghui Zhang^2,#^ ,Weiqi Dai^1^, Sainan Li^1^ ,Jiao Feng^1^,Jingjing Li^1^,Tong Liu^1^, Shizan Xu^3^,Wenwen Wang^1^,Xiya Lu^1^,Qiang Yu^3^,Kan Chen^1^,Yujing Xia^1^,Jie Lu^1^,Yingqun Zhou^1^,Xiaoming Fan^4,*^,Chuanyong Guo^1,*^**


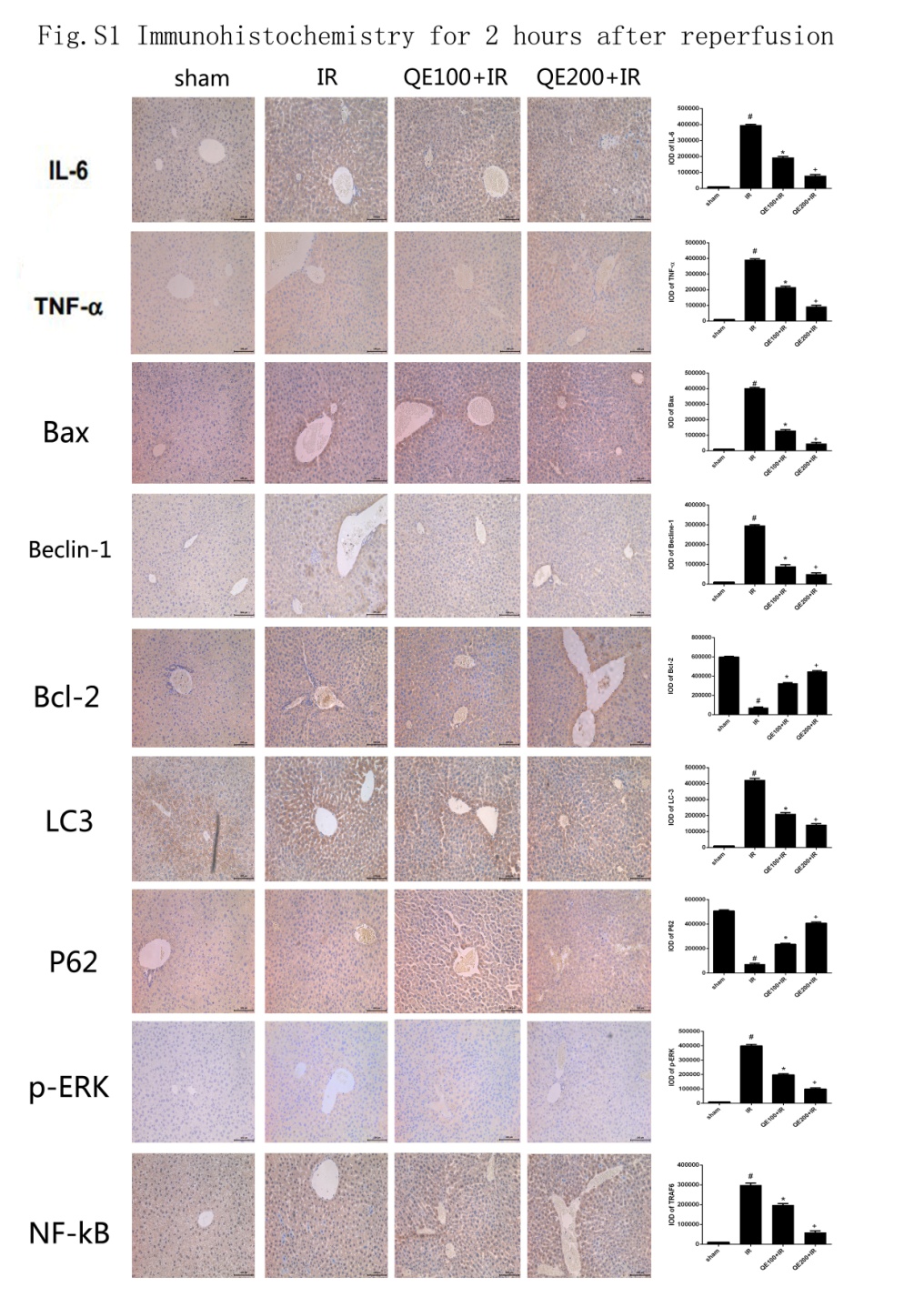

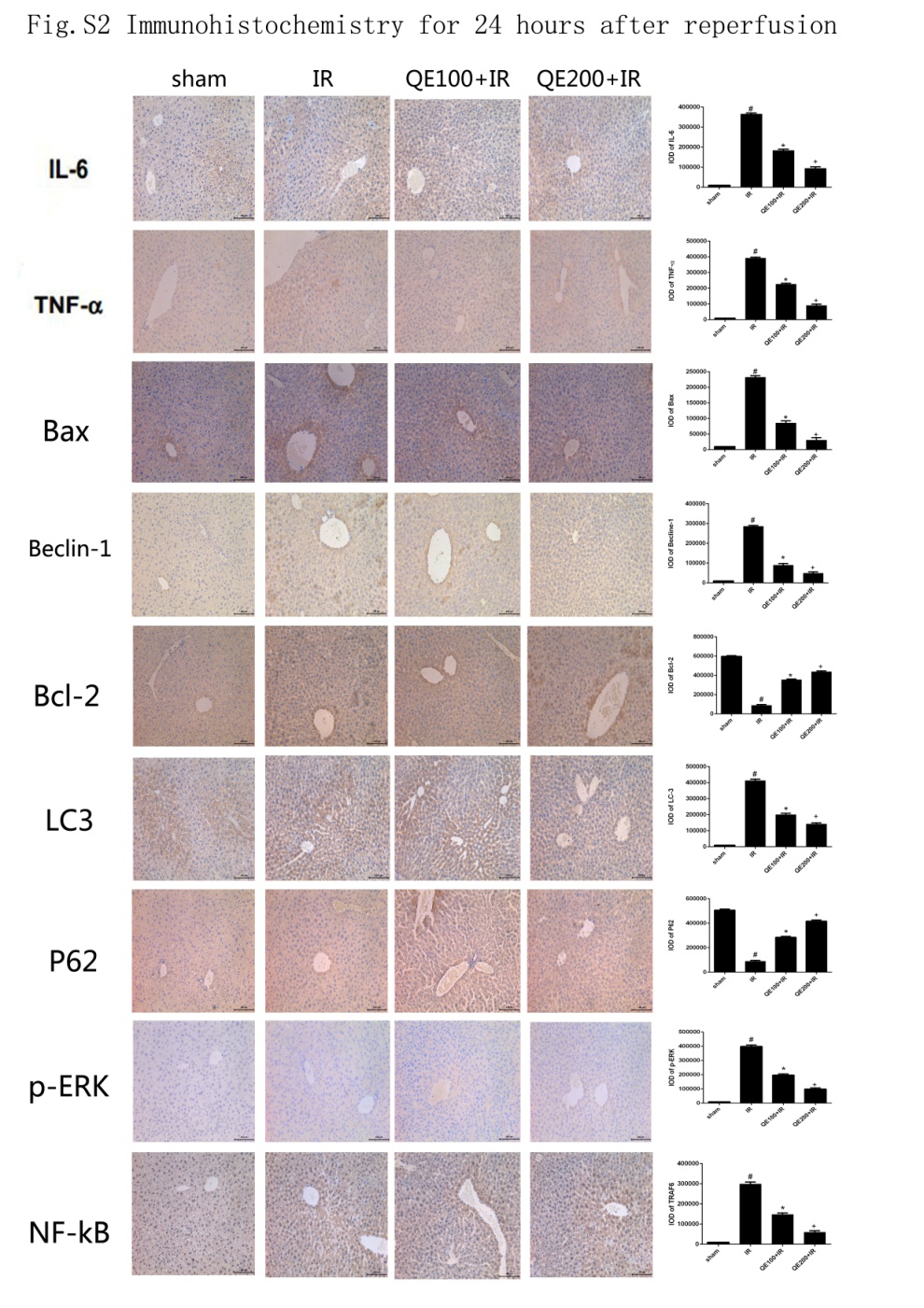

Supplement: Supplementary file 1 — Immunohistochemistry for 2 hours after reperfusion. Immunohistochemistry for 24 hours after reperfusion. [file 9724217.f1.docx]
